# Exogenous Melatonin Modulates Physiological Response to Nitrogen and Improves Yield in Nitrogen-Deficient Soybean (*Glycine max* L. Merr.)

**DOI:** 10.3389/fpls.2022.865758

**Published:** 2022-05-16

**Authors:** Huamei Wang, Chunyuan Ren, Liang Cao, Qiang Zhao, Xijun Jin, Mengxue Wang, Mingcong Zhang, Gaobo Yu, Yuxian Zhang

**Affiliations:** ^1^Key Laboratory of Soybean Mechanized Production, Ministry of Agriculture and Rural Affairs, Heilongjiang Bayi Agricultural University, Daqing, China; ^2^National Coarse Cereals Engineering Technology Center, Daqing, China

**Keywords:** soybean, melatonin, nitrogen deficiency, nitrogen metabolism, yield

## Abstract

Melatonin (MT) is a key plant growth regulator. To investigate its effect at different growth stages on the yield of soybean under nitrogen deficiency, 100 μM MT was applied to soybean supplemented with zero nitrogen (0N), low nitrogen (LN), and control nitrogen (CK) levels, during the plant vegetative growth (V3) and filling (R5) stages. This study revealed that the application of MT mainly enhanced the nitrogen fixation of plants by increasing the root nodule number and provided more substrates for glutamine synthetase (GS) under 0N supply. However, under the LN supply, more ammonium was assimilated through the direct promotion of nitrate reductase (NR) activity by MT. MT enhanced the activity of ammonium-assimilation-related enzymes, such as GOGAT and GDH, and the expression of their coding genes, promoted the synthesis of chlorophyll and amino acids, and increased the photosynthetic capacity under nitrogen deficiency. Exogenous MT directly upregulated the expression of genes involved in the photosynthetic system and stimulated dry-matter accumulation. Thus, MT alleviated the inhibitory effect of nitrogen deficiency on soybean yield. This mitigation effect was better when MT was applied at the V3 stage, and the seed weight per plant increased by 16.69 and 12.20% at 0N and LN levels, respectively. The results of this study provide a new theoretical basis to apply MT in agriculture to improve the resilience of soybean plants to low nitrogen availability.

## Introduction

Nitrogen is one of the essential nutrient elements for crop development, which directly or indirectly participates in a variety of physiological and biochemical processes, thus regulating crop yield (Arun et al., 2016; Han et al., 2016). As the main resource for fulfilling the world's protein needs, soybean (*Glycine max* L. Merr.) has the highest nitrogen requirement per unit of photosynthate among all food crops (Li et al., 2021). Approximately 80 kg of nitrogen is needed in aboveground dry matter for every ton of soybean seeds to be produced (Salvagiotti et al., 2008; Tamagno et al., 2017). This requirement is approximately three times higher than rice, wheat, corn, and other cereal crops per unit of grain (Barraclough et al., 2010; Setiyono et al., 2010; Yin et al., 2019). Although soybean has a unique nodule nitrogen fixation function which is different from other crops, the fixed nitrogen accounts for only 50–60% of demand, and hence, the insufficient supply of exogenous nitrogen is still an important factor restricting soybean growth (Grassini et al., 2015; Cafaro La Menza et al., 2020).

Nitrogen deficiency interferes with the normal carbon and nitrogen metabolism of soybean, by reducing photosynthesis and inhibiting the synthesis of amino acids and proteins (Xia et al., 2017; Liu et al., 2020). Insufficient nitrogen supply also affects plant morphogenesis, inhibits the increase of soybean leaf area, and changes the volume and total length of the root system (Li et al., 2018). In addition, a zero nitrogen condition significantly reduces yield by approximately 11% in the field, compared with the nitrogen supply suitable for optimal soybean yield (Cafaro La Menza et al., 2017; Tamagno et al., 2017). Because the V3 stage of soybean is the crucial stage of nodule development and plant morphogenesis, insufficient exogenous nitrogen supply at this stage can significantly limit nodulation and plant development (Gan et al., 2004; Purcell et al., 2014). During the filling stage (R5), nitrogen demand reaches its highest level, but the nitrogen fixation in root nodules and the ability of the root system to absorb nutrients begin to decrease (Ortez et al., 2019). Therefore, there is a close relationship between nitrogen absorption and utilization and yield formation of soybean in the V3 and R5 stages.

In recent years, to reduce the adverse effects of nitrogen application on the ecological environment, plant growth regulators that effectively improve the tolerance of crops to nitrogen deficiency are being explored (Du et al., 2020). In 1995, melatonin (MT) (N-acetyl-5-methoxytryptamine) was identified for the first time in plants (Dubbels et al., 1995). Since then, its role in plant development and growth has been widely investigated. MT can be used as a protective agent in many plants to improve stress resistance (Byeon and Back, 2014; Weeda et al., 2014; Kostopoulou et al., 2015; Liu et al., 2015; Gong et al., 2017). The chemical structure of MT is classified as an indole, and thus, its chemical function was considered to be similar to that of auxin (Tan et al., 2015; Back et al., 2016).

This resemblance has caused extensive studies into the effects of MT on plant growth, reproduction, and development (Nawaz et al., 2021). MT improves the photosynthetic capacity of plants and upgrades photosystems (PS) I and II—it increases nitrogen and chlorophyll content and elevates the levels of dissolvable proteins and Rubisco (Iqbal et al., 2021). MT can not only effectively facilitate root development but also delay leaf senescence, maintain chlorophyll content, and increase photosynthetic capacity and crop yield (Wei et al., 2015; Ding et al., 2017; Yang et al., 2017; Altaf et al., 2022). Under nitrogen stress, exogenous MT can help recover the yield of winter wheat by 16–23% (Qiao et al., 2019). The increase in biomass and yield of various crops may be due to MT promoting root development, thus indirectly regulating the absorption of nutrients (Zhang et al., 2017; Chen et al., 2018; Ren et al., 2019a) or directly regulating the ${\text{NH}}_{4}^{+}$/${\text{NO}}_{3}^{-}$ transporters and assimilation enzymes (Meng et al., 2020). Given the diverse roles of MT, Sun et al. (2020) proposed that it is a master regulator in plants. Nevertheless, the effective implementation of MT in agriculture practice is still limited (Nawaz et al., 2021). The regulation of MT on soybean yield under nitrogen deficiency has rarely been reported. This study investigates the regulatory mechanism of MT in soybean and whether the regulation pathways of MT in soybean are the same under different levels of nitrogen deficiency and at different growth stages.

As the V3 stage of soybean is key to the formation of vegetative bodies, and the R5 stage is the starting point for seed filling and expansion, attention was focused on the use of MT to regulate yield in these two growth stages. This study aimed to explore the impacts of MT on soybean low-nitrogen tolerance in the V3 and R5 stages and analyzed the mechanism of MT promoting soybean yield under nitrogen deficiency. The abilities closely related to soybean yield potential, including the absorption and utilization of nitrogen, nodule nitrogen fixation, and photosynthesis, were studied. The study has attempted to elucidate the biological function of MT in building the resilience of leguminous crops to nitrogen deficiency.

## Materials and Methods

### Plant Materials and Experimental Design

The study was conducted between May and October 2020, at the experimental base of the National Engineering and Technology Research Center for Grains in the High-tech Zone, Daqing City, Heilongjiang Province, China (46°39'N, 124°51'E). Soybean (*Glycine max* L. Merr.) seeds of uniform size were manually selected. The seeds were germinated in a germination box at 22°C for 2 days in the dark. Seedlings with consistent growth were chosen and transplanted at a density of three buds per 33 × 26 cm bucket that was filled previously with fine river sand. The sand was first rinsed with tap water to remove turbidity and then rinsed three times with distilled water. Six 1 cm holes were drilled through the base of each bucket and gauze was placed at the base inside the bucket before filling with sand. Plants were watered with 0.5 L of distilled water per bucket per day until the opposite leaves were fully unfolded.

After the full expansion of opposite leaves, the plants were randomly divided into two groups, which were treated with low nitrogen (LN) and control nitrogen (CK). A nitrogen (N) concentration of 7.5 mM, contained in 1/2 Hoagland nutrient solution, was used as a control (Li et al., 2017; Qiao et al., 2019). CaCl_2_ and KCl were used to supplement insufficient elements (Wang et al., 2019) (see Supplementary Table 1 for formula of nutrient solution). Each bucket was treated with 0.5 L of solution. To prevent the accumulation of salt in the sand culture, buckets were rinsed thoroughly with distilled water every 3 days. The soybeans were treated with 100 μM MT when they developed to V3 or R5 stage. Physiological metabolism-related indices, including nitrogen assimilation, related enzyme activity, net photosynthetic rate, chlorophyll content, and soluble sugar content, were measured at 6, 12, 18, and 24 days after MT treatment (Supplementary Figures 1, 2), and the dry matter accumulation of plants and nodules at each investigated growth stage after MT treatment was analyzed (Supplementary Table 2). The time duration with the most obvious difference between MT treatment and non-MT treatment under LN was selected as the reference for sampling time in the 2nd year.

The experiment was conducted with three nitrogen concentration treatments (i.e., 0N, LN, and CK, Supplementary Table 1) in 2021. At three nitrogen levels, MT was sprayed at V3 or R5 stage or not applied, and 9 treatments were obtained. Samples were collected 12 days after the MT application (based on the results of the screening experiment in 2020) for the determination of physiological indices. The functional leaf samples were collected 24 h after MT treatment and stored at −80°C for quantitative reverse transcription PCR (RT-qPCR) analysis. The functional leaf samples were directly determined or wrapped in tin foil and stored at −20°C for physiological and biochemical analysis, and the remaining plants were used to determine the nitrogen and ureides content in each organ of the plant after maturity. Three repeated values were recorded for each physiological and biochemical index and averaged (from three mixed samples) for those in the same container, and 15 repeated values were recorded for the determination of yield components.

### Measurements of Nitrogen-Assimilating Enzyme Activity

The tissue of fresh functional leaves was weighed into 0.5 g portions to determine enzyme activity. Nitrate reductase (NR: EC 1.7.1.3) activity was measured according to the method of Sherrard and Dalling (1979). Glutamine synthetase (GS: EC 6.3.1.2) enzyme activity was determined according to Zhang et al. (2017). The activities of glutamate synthase (GOGAT: EC 1.4.7.1) and glutamate dehydrogenase (GDH: EC 1.4.1.2) were determined according to the method of Lin and Kao (1996).

### Measurement of Inorganic Nitrogen Content

Fresh leaf samples were weighed to 1 g (repeated three times). The extract used for biochemical analysis was prepared using the method of Oliveira et al. (2013). The content of ${\text{NO}}_{3}^{-}$ was determined spectrophotometrically at 410 nm by the salicylic acid method described by Cataldo et al. (1975). The level of ${\text{NO}}_{2}^{-}$ was determined according to the method described by Hageman and Reed (1980).

### Measurement of Free Amino Acid and Soluble Protein Content

The tissue stored at −20°C was divided into 0.5 g samples. The content of soluble protein was determined by spectrophotometry according to the method described by Cao et al. (2019); the content of total free amino acids was determined by using ninhydrin reagent (Moore and Stein, 1954).

### Measurement of Total Nitrogen Content

The content of total nitrogen in plant tissues was measured by the Kjeldahl method (Zhang et al., 2010). The mature samples dried at 105°C were ground into powder. The samples were weighed to 0.1 g, digested with H_2_SO_4_-H_2_O_2_, and then N content was determined by a Kjeldahl nitrogen determination instrument. Each treatment was repeated at least three times.

### Measurement of Soybean Nodulation and Nitrogen Fixation Ability

The manual counting method was used to calculate the number of root nodules per plant of soybean. The fresh nodules were dried at 105°C for 0.5 h, then dried in an oven at 80°C to constant weight, and the dry weight of the nodules was measured. Nodule nitrogenase activity was evaluated by measuring hydrogen production in living soybean nitrogen fixation tissues using a nitrogen fixation analyzer (Q-BOXNFILP) to determine the rate of nitrogen change. According to the method described by Zhang et al. (2018), the content of ureides in the dried organ at the mature stage was determined.

### Measurement of Chlorophyll Content and gas Exchange Parameters

The content of chlorophyll a (Chl a), chlorophyll b (Chl b), and total chlorophyll (total Chl) was determined by the method of Cao et al. (2019). The net photosynthesis rate (Pn), transpiration rate (Tr), stomatal conductance (Gs), and intercellular CO_2_ concentration (Ci) of the 2nd leaf from the top of the main stem were measured using a Li-6400 photosynthesis system (Li-6400, LICOR, Lincoln, NE, USA) equipped with a red-blue LED artificial light source leaf chamber using the method described by Wang et al. (2021a). During the measurement, photosynthetically active radiation (PAR) was 1,000 μmol/m^2^ s, atmospheric CO_2_ concentration was 400 μmol/mol, leaf temperature was 25°C, and the relative humidity was ~25%. All measurements were made on sunny days between 09:00 and 16:00 (Liu and Li, 2016).

### Total RNA Extraction and Gene Expression (RT-qPCR) Analysis

Total RNA of the functional leaf sample (50–100 mg) was extracted using an Axygen reagent kit (AxyPrep, USA) and reverse-transcribed according to the manufacturer's instructions (Prime Script RT Enzyme Mix I for TB Green qPCR, Takara, China). The gene-specific primers were designed with Primer Premier 5.0 (Supplementary Table 3), and RT-qPCR was performed using a 10 μl mixture (5 μl TB Green Premix Ex Taq II; 3 μl distilled water; 0.5 μl PCR Forward Primer; 0.5 μl PCR Reverse Primer; 1 μl cDNA) according to the manufacturer's instructions (CFX96 Real-Time PCR Detection System, Takara, China). A two-step PCR method was used with the following conditions: pre-denaturation at 95°C for 30 s, and 40 cycles at 95°C for 5 s and 60°C for 30 s. The specialist software Bio-Rad iQ5 was used to collect data. Actin (gene ID: *Glyma18g52780*) was adopted as an internal reference for the normalization of gene expression levels. In the RT-qPCR analysis, samples of two plants from each pot treated with MT for 24 h were mixed as a single replicate, and three pots per treatment were considered as three biological replicates. Each biological replicate had three technical replicates.

### Measurement of Yield and Composition

Five pots of soybean under each treatment were randomly selected at harvest for the assessment of the number of pods per plant, the number of grains per plant, total grain weight per plant, and hundred-grain weight.

### Statistical Analyses

The experiment was conducted in a randomized design. The data obtained were analyzed by Microsoft Excel 2017. LSD and Duncan's multiple range test (*P* ≤ 0.05) were performed using SPSS 20.0 (SPSS Inc., Chicago, IL, USA). The charts including the PCA Biplot were formed with Origin 2019 software.

## Results

### Effects of Exogenous Melatonin on Enzyme Activity Related to Nitrogen Assimilation

**Figure 1:**
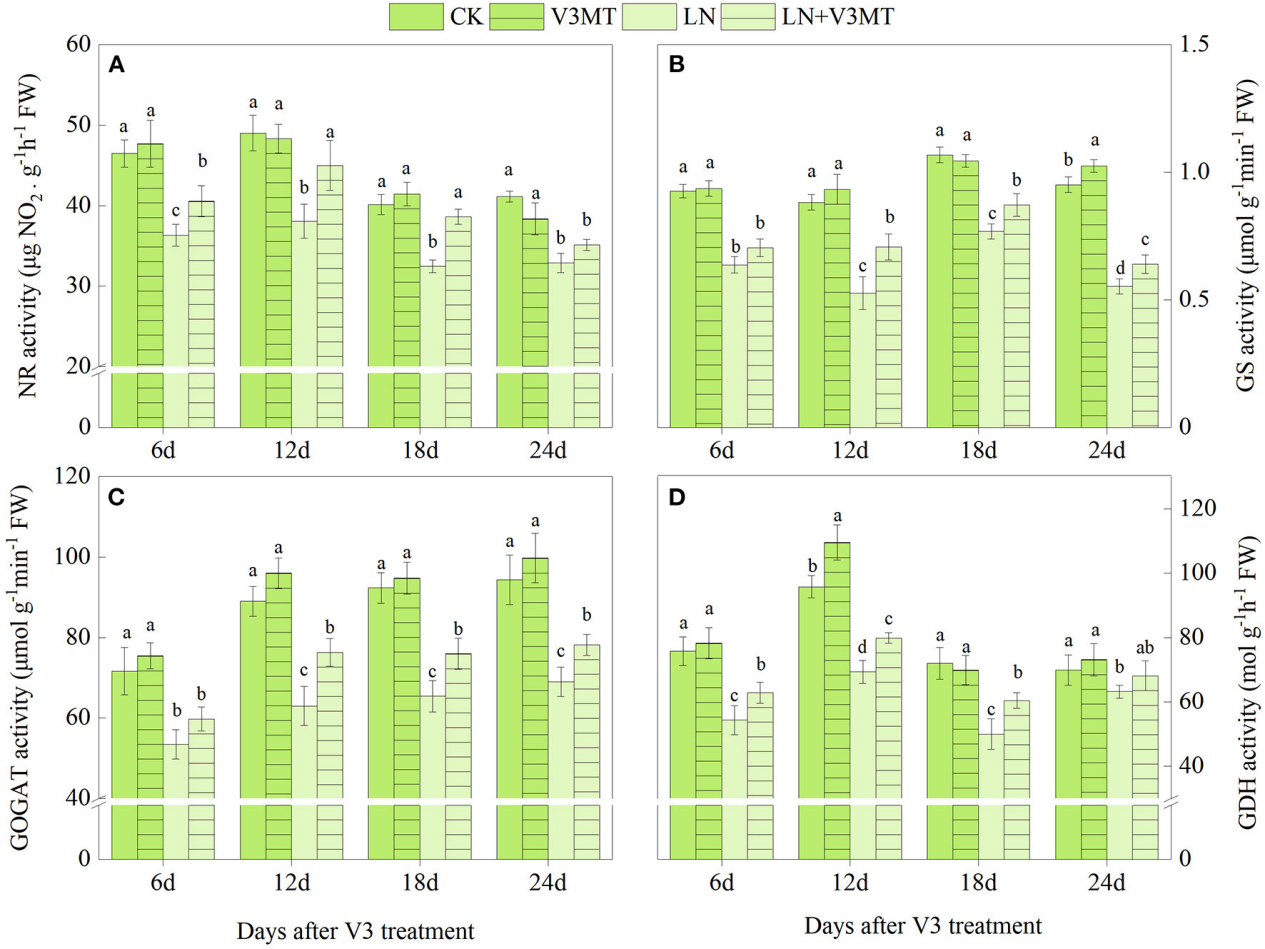
The effect of spraying melatonin at V3 stage on the activities of nitrate reductase (NR, **A**), glutamine synthetase (GS, **B**), glutamate synthase (GOGAT, **C**), and glutamate dehydrogenase (GDH, **D**) in plant leaves under two N levels. CK, control nitrogen; V3MT, melatonin application at V3 stage under control nitrogen level; LN, low nitrogen; LN+V3MT, melatonin application at V3 stage under low nitrogen level. The values were presented as mean ± SE (*n* = 3). Different letters in one measuring group indicate statistically significant differences when *P* ≤ 0.05.

**Figure 2:**
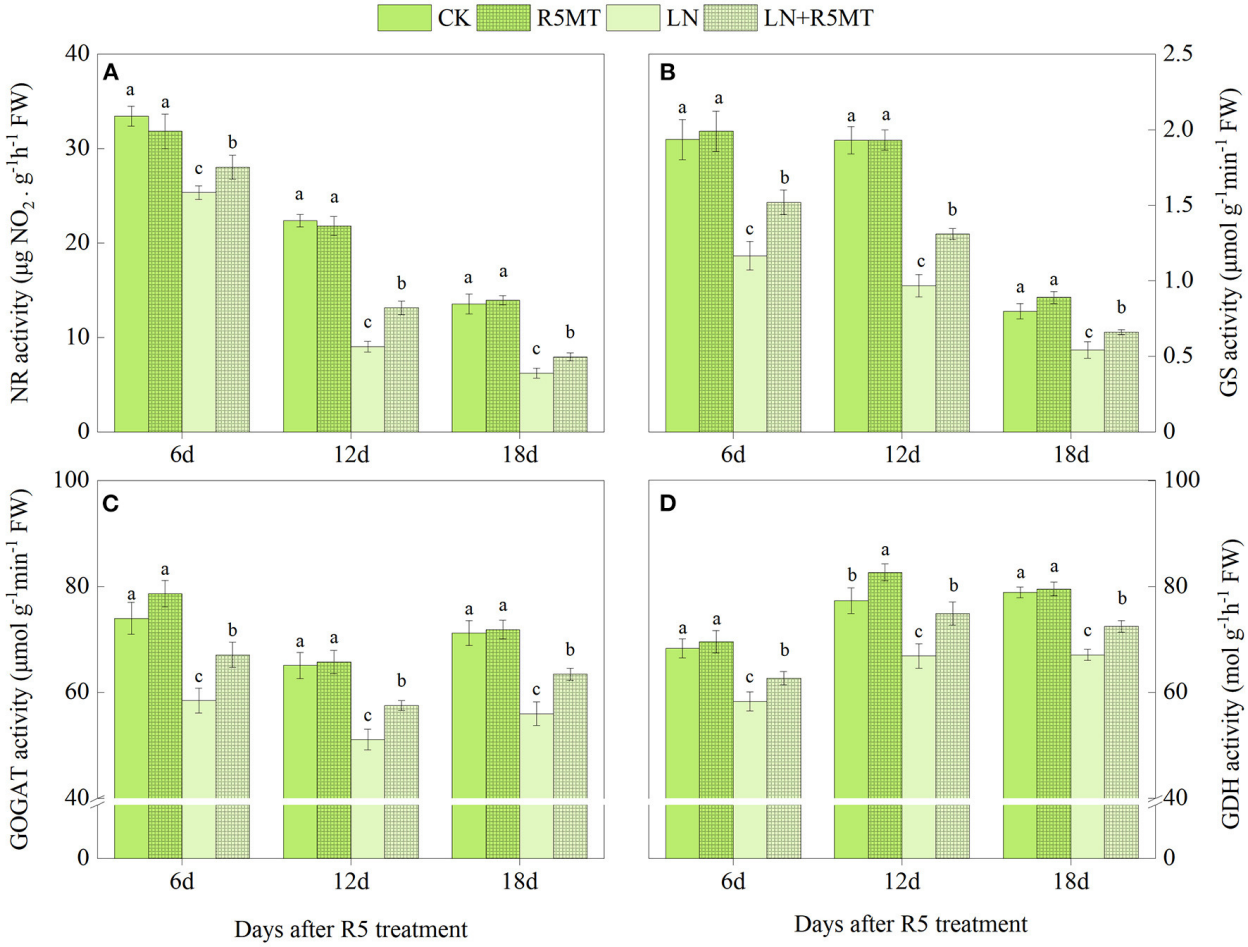
The effect of spraying melatonin at R5 stage on the activities of nitrate reductase (NR, **A**), glutamine synthetase (GS, **B**), glutamate synthase (GOGAT, **C**), and glutamate dehydrogenase (GDH, **D**) in plant leaves under two N levels. CK, control nitrogen; R5MT, melatonin application at R5 stage under control nitrogen level; LN, low nitrogen; LN+R5MT, melatonin application at R5 stage under low nitrogen level. The values were presented as mean ± SE (*n* = 3). Different letters in one measuring group indicate statistically significant differences when *P* ≤ 0.05.

As shown in Figures 1, 2, the activities of NR, GS, GOGAT, and GDH treated with LN were significantly lower than those in the CK group. The application of MT effectively promoted the activity of these nitrogen assimilation enzymes under LN level. At 12 days after spraying MT, the enhancement of enzyme activity in LN+V3MT was the highest, and the activity levels of NR, GS, GOGAT, and GDH increased by 18.20, 34.42, 21.16, and 15.22%, respectively, compared with those in LN (Figure 1). Moreover, MT spraying at R5 stage showed a similar result to V3 stage, i.e., compared with LN, MT addition had the largest increase in nitrogen metabolic enzymes at day 12 after MT treatment (Figure 2).

**Figure 3:**
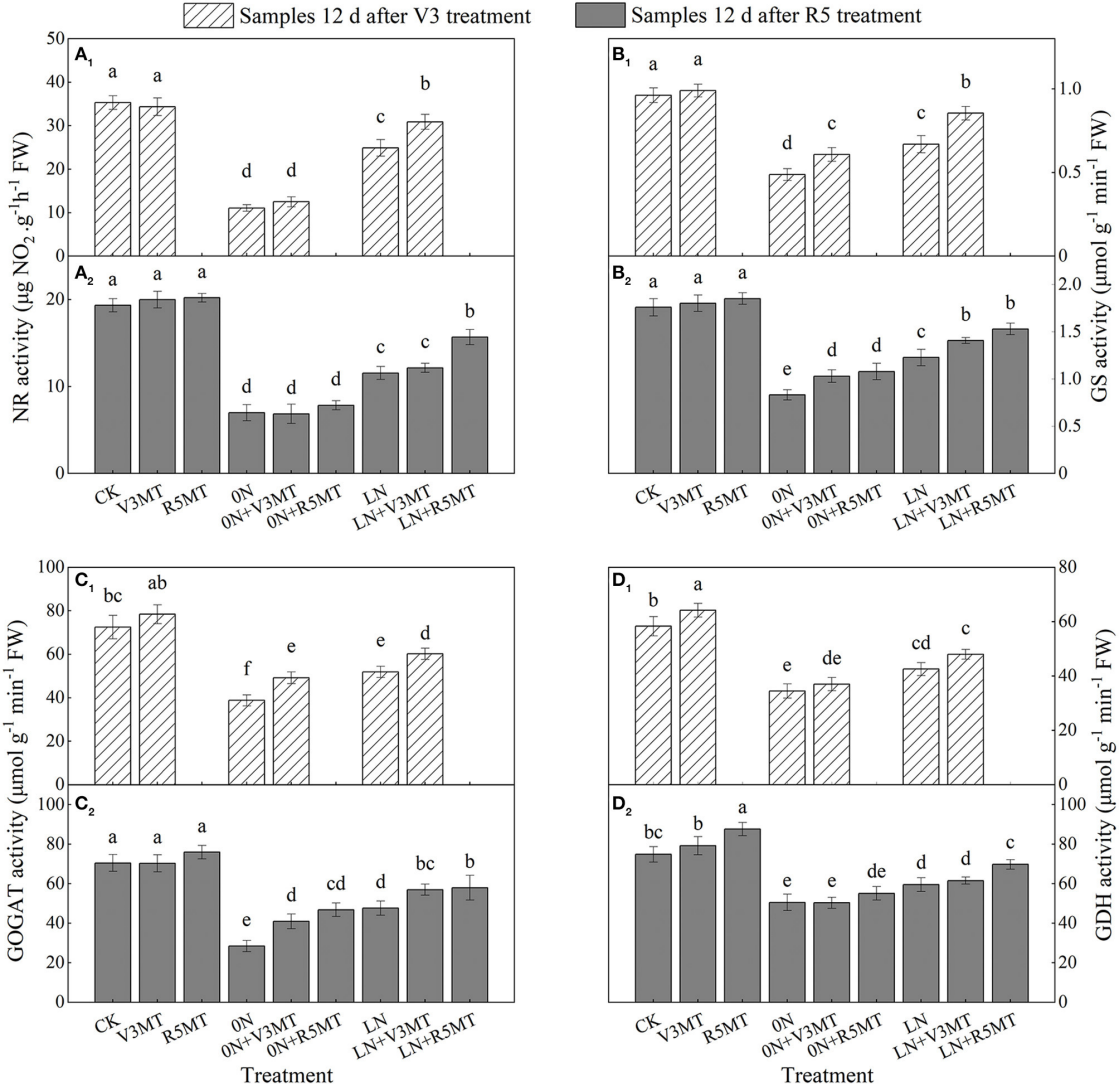
The effect of exogenous melatonin (V3 and R5) on the activities of nitrate reductase (NR, **A**), glutamine synthetase (GS, **B**), glutamate synthase (GOGAT, **C**), glutamate dehydrogenase (GDH, **D**) in plant leaves under three N levels. CK, control nitrogen; V3MT, melatonin application at V3 stage under control nitrogen level; R5MT, melatonin application at R5 stage under control nitrogen level; 0N, zero nitrogen; 0N+V3MT, melatonin application at V3 stage under zero nitrogen level; 0N+R5MT, melatonin application at R5 stage under zero nitrogen level; LN, low nitrogen; LN+V3MT, melatonin application at V3 stage under low nitrogen level; LN+R5MT, melatonin application at R5 stage under low nitrogen level. The values were presented as mean ± SE (*n* = 3). Different letters in one measuring group indicate statistically significant differences when *P* ≤ 0.05.

Compared with the CK, MT significantly enhanced the activity of GDH, while the activity of nitrogen metabolism enzymes decreased under 0N and LN treatments (Figure 3). Significant increases in the activity of GS, GOGAT, and GDH were observed under MT application for both nitrogen deficiency treatments, and the promoting effect was more obvious under 0N (Figures 3B–D). However, the alleviation effect of MT on the inhibited NR activity was mainly observed in the low nitrogen supply group, in which the NR activity in LN+V3MT and LN+R5MT increased by 24.08 and 35.59%, respectively, compared with that in LN at 12 days after MT treatment (Figure 3A). Furthermore, 12 days after MT treatment at R5 stage, the GS and GOGAT activities of 0N+V3MT and LN+V3MT were markedly higher than those of the treatment without MT under the corresponding nitrogen level. Specifically, after the MT treatment, GS activity increased by 23.75 and 14.59%, and GOGAT activity was enhanced by 43.86 and 19.58%, respectively, when compared with the corresponding nitrogen level (Figures 3B_2_,C_2_).

### Effects of Exogenous Melatonin on the Contents of Nitrate and Ammonium

**Figure 4:**
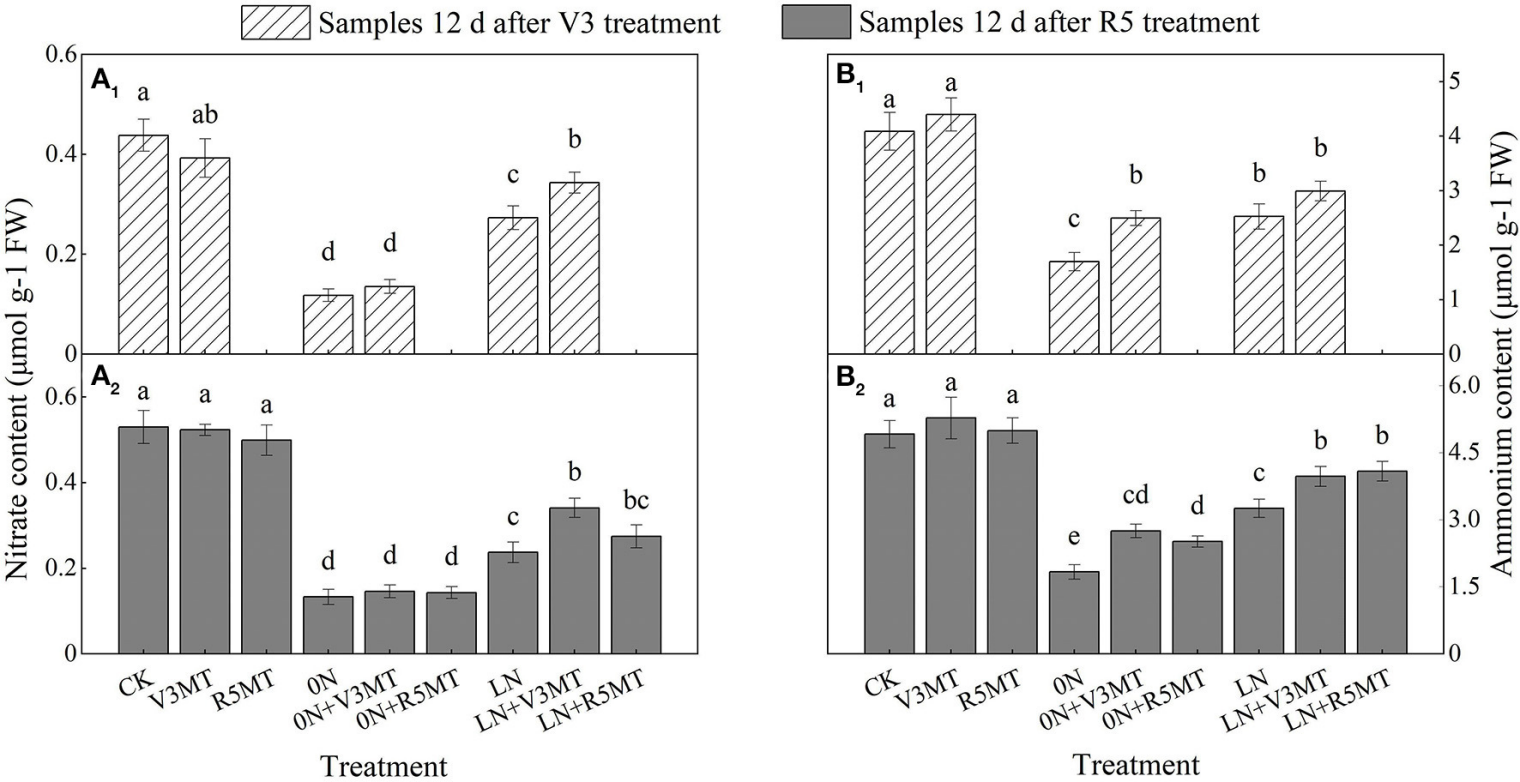
The effect of exogenous melatonin (V3 and R5) on the content of nitrate **(A)** and ammonium **(B)** in plant leaves of under three N levels. The abbreviated meaning of each treatment is the same as in Figure 3. The values were presented as mean ± SE (*n* = 3). Different letters in one measuring group indicate statistically significant differences when *P* ≤ 0.05.

Differences in inorganic nitrogen contents were not evident between CK and V3MT/R5MT. Regulation by MT of ${\text{NO}}_{3}^{-}$ and ${\text{NH}}_{4}^{+}$ in functional leaves was mainly reflected in 0N and LN levels (Figure 4). As a result of V3 application, MT primarily promoted the accumulation of ${\text{NO}}_{3}^{-}$ at the low nitrogen level. At the two sampling time points, the ${\text{NO}}_{3}^{-}$ content of LN+V3MT was 25.61 and 43.68% higher than that of LN, respectively, but lower than that of the CK treatment (Figure 4A). In contrast, the contribution of exogenous MT to ${\text{NH}}_{4}^{+}$ accumulation was more significant at 0N level (Figure 4B).

### Effects of Exogenous Melatonin on the Contents of Soluble Protein and Free Amino Acids

**Figure 5:**
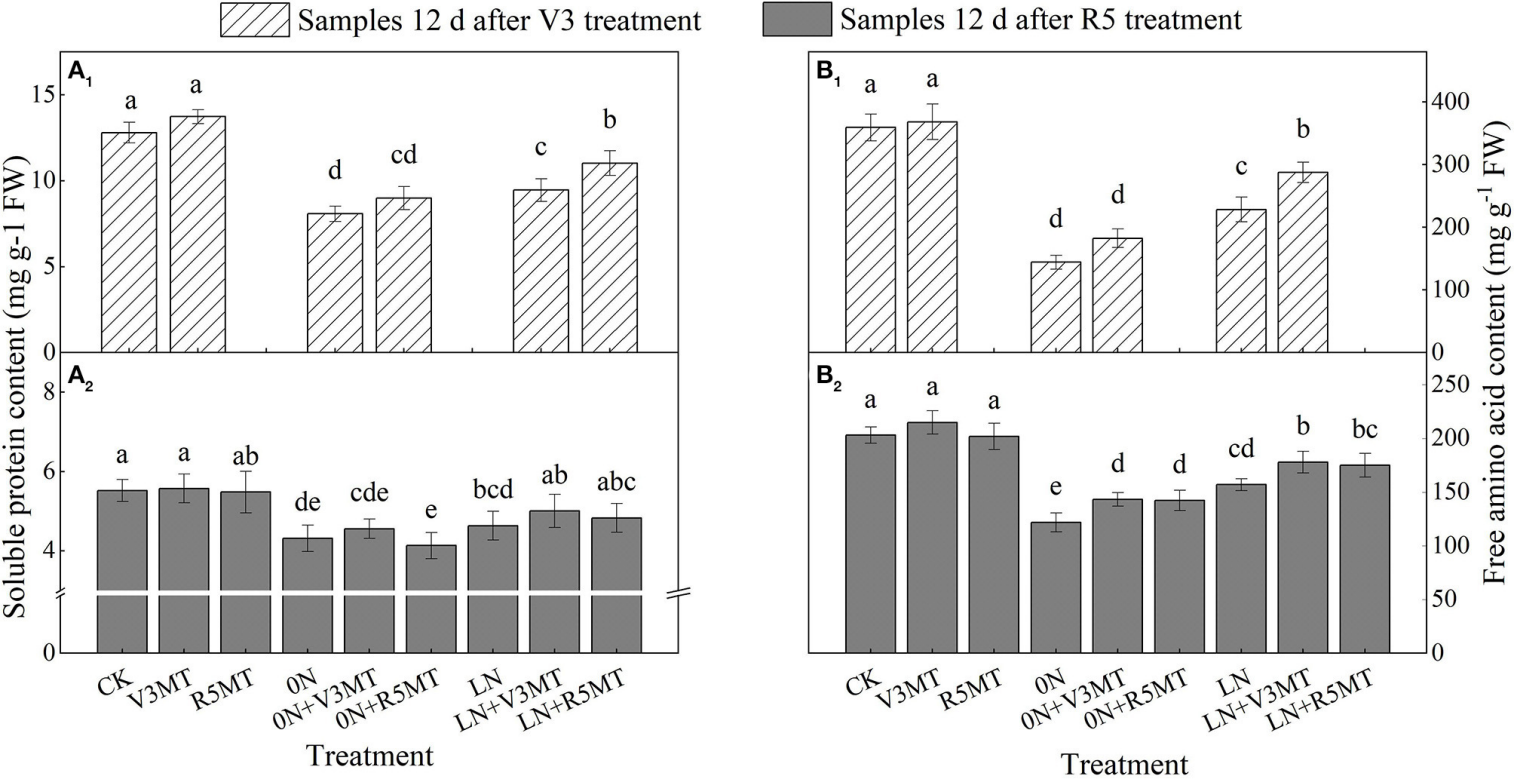
The effect of exogenous melatonin (V3 and R5) on the content of soluble protein **(A)** and free amino acid **(B)** in plant leaves under three N levels. The abbreviated meaning of each treatment is the same as in Figure 3. The values were presented as mean ± SE (*n* = 3). Different letters in one measuring group indicate statistically significant differences when *P* ≤ 0.05.

Soluble protein and free amino acid contents were affected by insufficient nitrogen supply, with the levels lower than those in the CK group (Figure 5). Compared with LN, the content of soluble protein was enhanced by 16.64% when MT was applied at V3 stage (Figure 5A_1_). The application of MT at R5 stage had an insignificant effect on soluble protein content (Figure 5B_1_). The content of free amino acids was raised in both MT application stages compared with their corresponding nitrogen levels. Moreover, the contents of free amino acids in functional leaves treated with 0N+V3MT and LN+V3MT were still significantly higher than those treated with 0N and LN, respectively, at 12 days after R5 spraying (Figure 5B_2_).

### Effects of Exogenous Melatonin on the Number, Dry Weight, and Nitrogenase Activity of Root Nodules

**Figure 6:**
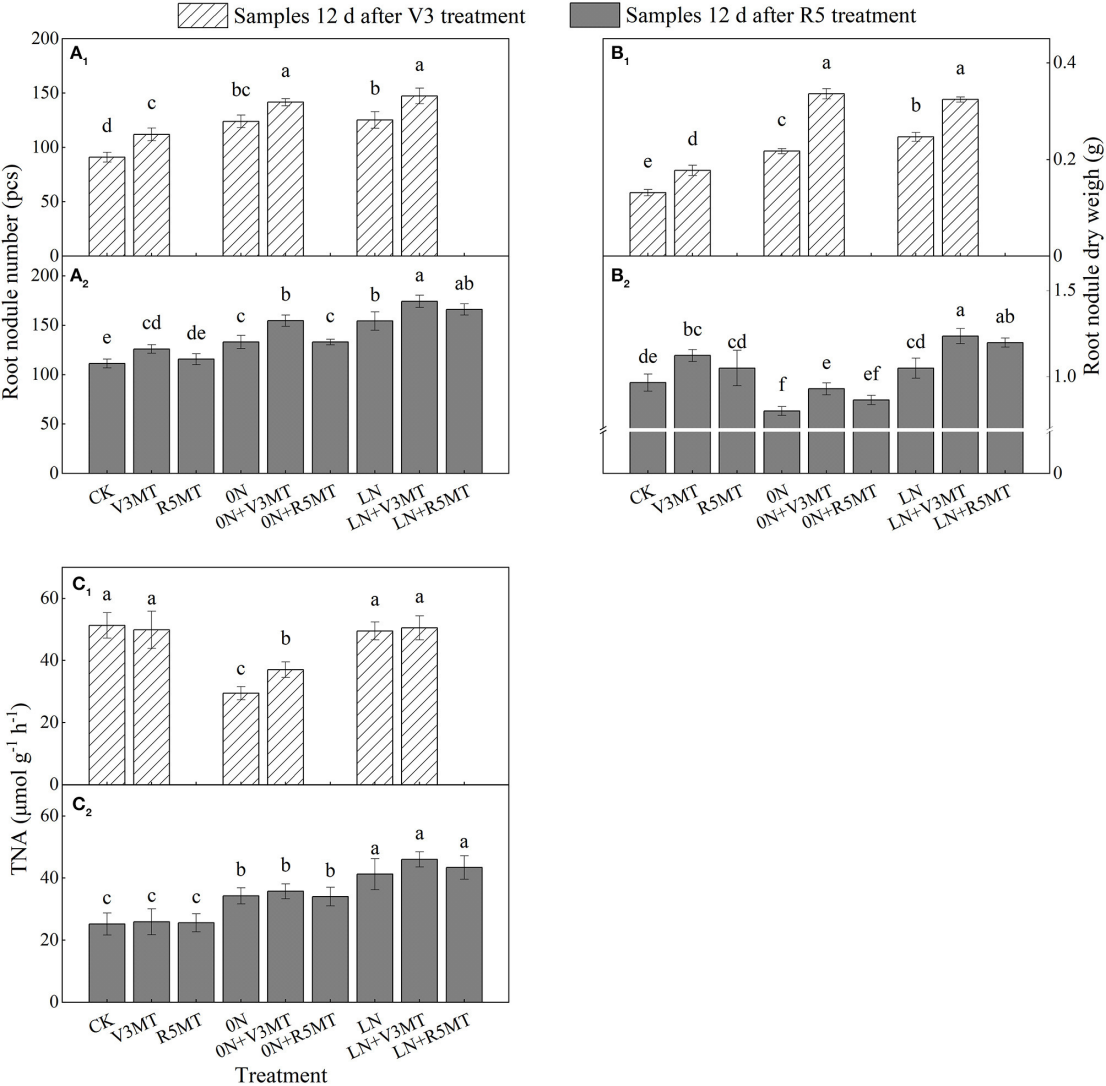
The effect of exogenous melatonin (V3 and R5) on the number **(A)**, dry weight **(B)**, and nitrogenase activity **(C)** of root nodules under three N levels. The abbreviated meaning of each treatment is the same as in Figure 3. The values were presented as mean ± SE (*n* = 3). Different letters in one measuring group indicate statistically significant differences when *P* ≤ 0.05.

Melatonin increased the nodule number and nodule dry weight by 23.08 and 35.03%, respectively, 12 days after V3 stage treatment (Figures 6A_1_,B_1_) when compared with the CK. Notably, the activity of nitrogenase in root nodules was only promoted by MT application at V3 stage under 0N level (Figure 6C). In the condition of nitrogen deficiency (0N and LN), the number, dry weight, and nitrogen fixation ability of root nodules were higher than those of CK, and the optimal parameters were reflected in the LN level (Figure 6). The MT treatment at V3 stage further increased the number and dry weight of nodules under nitrogen deficiency. The number and dry weight of nodules treated by LN+V3MT were highest, even 12 days after MT was applied in R5 stage, and were 56.59 and 28.07% more than that of CK, respectively (Figures 6A_2_,B_2_). However, the application of MT at R5 stage only affected the dry weight of nodules at low nitrogen levels, increasing it by 14.11% compared with LN (Figure 6B_2_).

### Effects of Exogenous Melatonin on Ureides Content of Each Organ

**Figure 7:**
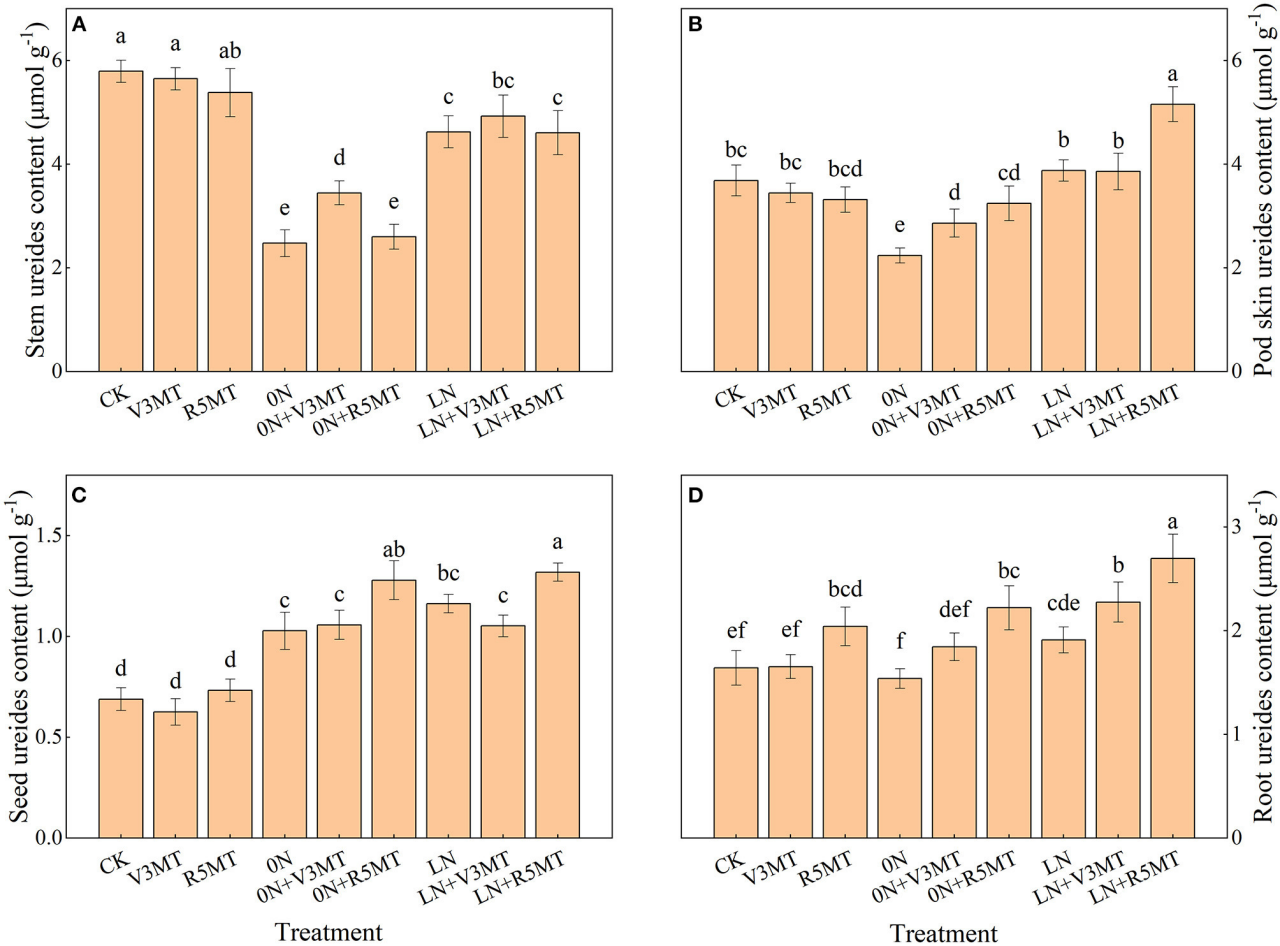
The effect of exogenous melatonin (V3 and R5) on ureide content of stem **(A)**, pod skin **(B)**, seed **(C)**, and root **(D)** under three N levels. The abbreviated meaning of each treatment is the same as in Figure 3. The values were presented as mean ± SE (*n* = 3). Different letters in one measuring group indicate statistically significant differences when *P* ≤ 0.05.

Exogenous MT application at R5 stage (R5MT) resulted in more ureides content in roots at harvest when compared with the CK treatment (Figure 7D). Nitrogen deficiency caused the transfer of ureide to seed and reduced the accumulation of ureides in stem and pod skin at harvest (Figures 7A–C). Regardless, when seedlings were treated with MT at V3 stage, the ureide accumulation in the stem, pod skin, and root increased under 0N level (Figures 7A,B,D). In contrast, the MT treatment at R5 stage mainly promoted the ureides content in pod skin, grain, and root both under 0N and LN levels (Figures 7B–D). The ureides content in seeds under 0N+R5MT and LN+R5MT conditions was 0.59 and 0.63 μmol/g higher than that of the control, respectively (Figure 7C).

### Effects of Exogenous Melatonin on Nitrogen Content of Each Organ

**Figure 8:**
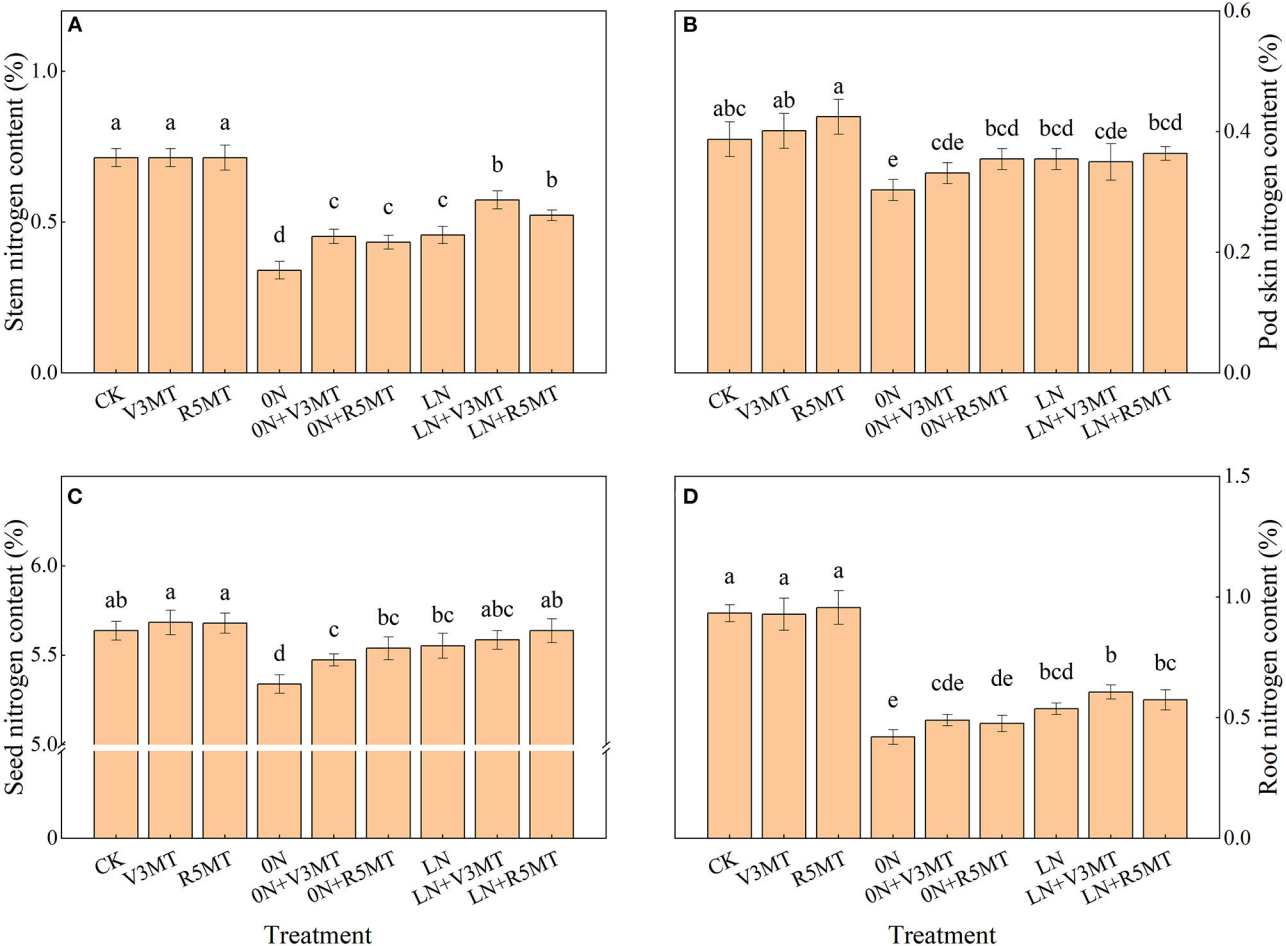
The effect of exogenous melatonin (V3 and R5) on the nitrogen content of stem **(A)**, pod skin **(B)**, seed **(C)**, and root **(D)** under three N levels. The abbreviated meaning of each treatment is the same as in Figure 3. The values were presented as mean ± SE (*n* = 3). Different letters in one measuring group indicate statistically significant differences when *P* ≤ 0.05.

The nitrogen content of each organ at 0N level during harvest was decreased when compared with CK, while the difference in its accumulation between LN and CK was mainly reflected in the stem and root (Figures 8A,D). When MT was applied alone at V3 or R5 stage of soybean, the change in nitrogen content was insignificant in comparison with CK. However, the increases in nitrogen content in the stem of 0N+V3MT and 0N+R5MT were 32.88 and 27.40%, respectively, when compared with 0N (Figure 8A). Likewise, increases in the seed for those same treatment groups were 2.53 and 3.76%, respectively (Figure 8C). Furthermore, the application of MT at V3 and R5 under LN treatment increased the nitrogen content of stems by 25.51 and 14.29%, respectively (Figures 8A,B).

### Effects of Exogenous Melatonin on Chlorophyll Content and Photosynthetic Ability

**Figure 9:**
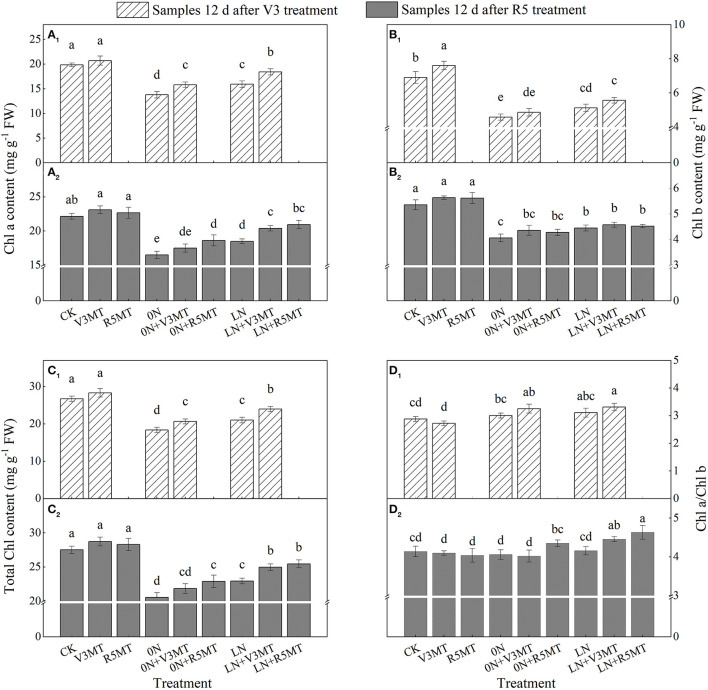
The effect of exogenous melatonin (V3 and R5) on the content of Chl a **(A)**, Chl b **(B)**, total Chl **(C)**, and the ratio of Chl a/Chl b **(D)** in plant leaves under three N levels. The abbreviated meaning of each treatment is the same as in Figure 3. The values were presented as mean ± SE (*n* = 3). Different letters in one measuring group indicate statistically significant differences when *P* ≤ 0.05.

Compared with CK, insufficient nitrogen supply significantly suppressed the levels of Chl a and Chl b, and the inhibitory affect was more evident in 0N (Figures 9A–C). The degree of decrease in Chl a content was lower than that of Chl b both in 0N and LN, so the ratio of Chl a/Chl b increased when compared with CK at the seedling stage (Figure 9D). MT alone at V3 stage was beneficial to increase the content of Chl b that plays an auxiliary role. However, the application of MT markedly alleviated the inhibition effect of N deficiency both at V3 and R5 stages, particularly on the content of Chl a (Figure 9A). Notably, MT treatment at R5 stage further increased the ratio of Chl a/Chl b, which showed that the increase of Chl a content was greater than Chl b in 0N+R5MT and LN+R5MT treatments compared with corresponding nitrogen treatments (Figure 9D_2_).

**Figure 10:**
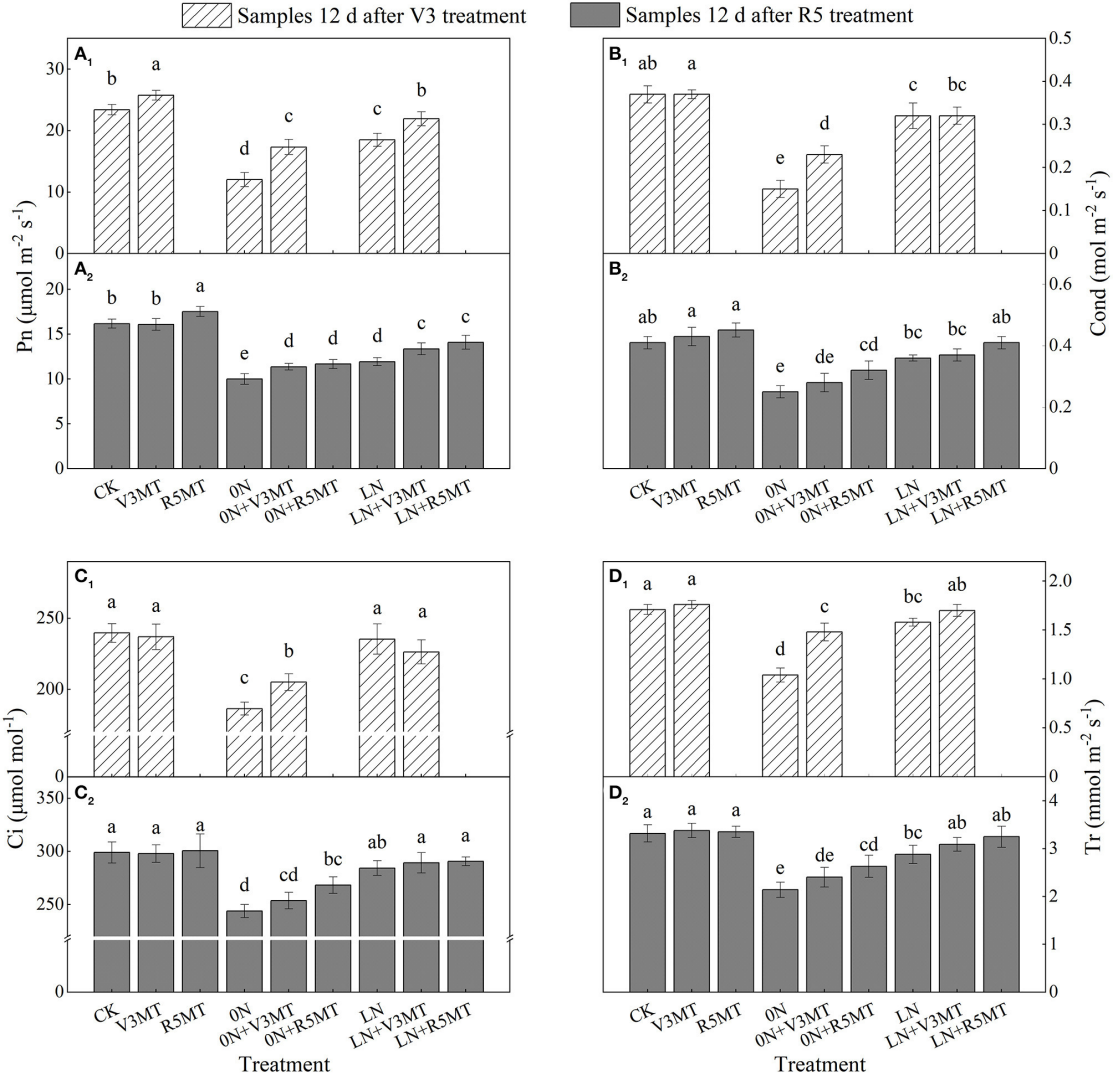
The effect of exogenous melatonin (V3 and R5) on the net photosynthetic rate (Pn, **A**), stomatal conductance (cond, **B**), intercellular carbon dioxide concentration (Ci, **C**), and transpiration rate (Tr, **D**) in plant leaves under three N levels. The abbreviated meaning of each treatment is the same as in Figure 3. The values were presented as mean ± SE (*n* = 3). Different letters in one measuring group indicate statistically significant differences when *P* ≤ 0.05.

The leaf gas exchange parameters were severely inhibited when nitrogen supply was reduced (0N and LN), particularly in 0N conditions (Figure 10). The exogenous MT at V3 and R5 stages enhanced Pn under all nitrogen levels including the control nitrogen level (Figure 10A). When MT was applied in V3 stage under LN condition, Pn returned to the same level as the control (Figure 10A_1_). However, spraying MT at V3 stage significantly promoted Cond, Ci, and Tr under 0N level only at 12 days after application (Figures 10B_1_–D_1_). On the 12th day after R5 treatment, the Pn of 0N+V3MT was significantly higher than that of 0N by 13.67%, while there was no distinction in Cond, Ci, and Tr between 0N+V3MT and 0N (Figures 10B_2_–D_2_). In contrast, for 0N+R5MT, the photosynthetic correlation indices Pn, Cond, Ci, and Tr increased by 16.67, 25.43, 9.97, and 22.90%, respectively, in comparison with 0N (Figures 10A_2_–D_2_).

### Effects of Exogenous Melatonin on the Expression of Genes Related to Nitrogen Metabolism and Photosynthesis

**Figure 11:**
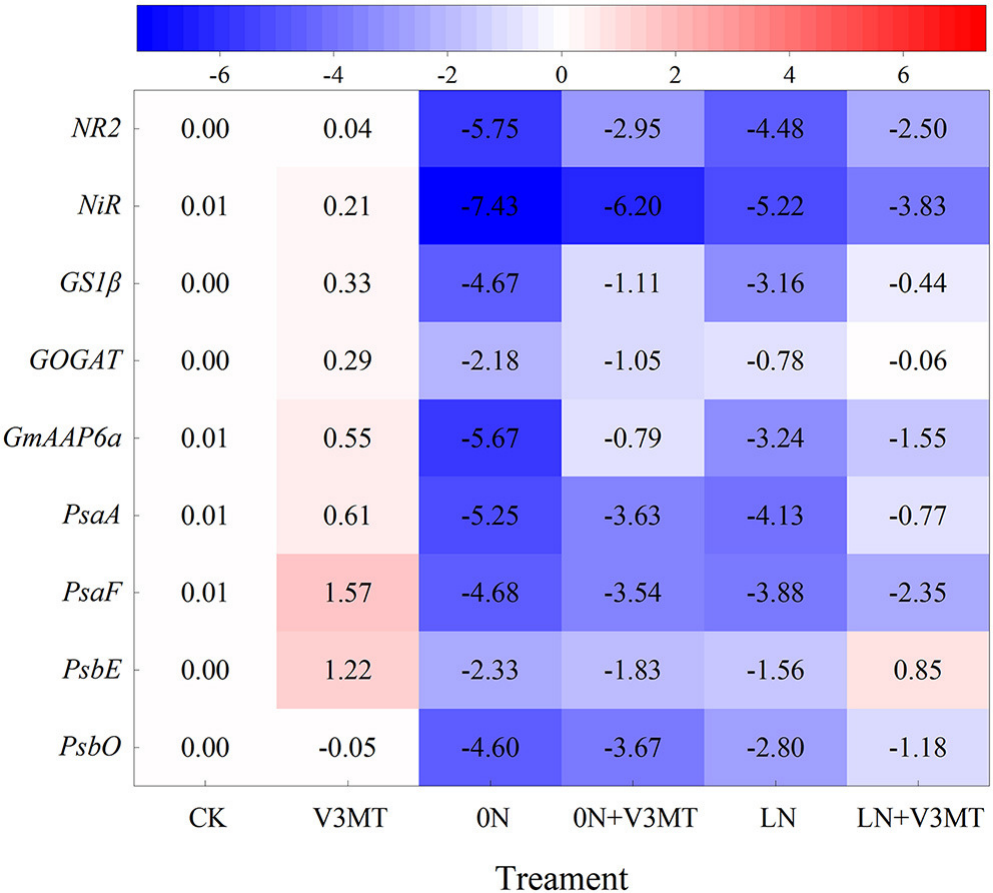
The effect of exogenous melatonin on the relative expressions of nitrate reductase gene (*NR2*), nitrite reductase gene (*NiR*), glutamine synthetase gene (*GS1*β), glutamate synthase gene (*GOGAT*), gene related to amino acid assignment (*GmAAP6a*), and genes related to photosynthesis (*PsaA, PsaF, PsbE, PsbO*) in plant leaves under three N levels. The abbreviated meaning of each treatment is the same as in Figure 3. Taking the average value of the actin gene and CK expression as reference, the values were the average of 2^−(ΔΔCT)^ presented.

Gene expression related to MT-induced nitrogen metabolism and photosynthesis is presented in Figure 11. When MT was applied alone, the expression levels of *PsaF* and *PsbE* related to photosynthetic system I (PSI) and photosynthetic system II (PSII) were 1.57 and 1.22 times higher, respectively, than that of CK. Under nitrogen deficiency, the genes effectively upregulated by MT included not only those encoding PSI (*PsaA, PsaF*) and PSII (*PsbE, PsbO*) but also those encoding nitrogen assimilation enzymes (*NR2, NiR, GS1*β, and *GOGAT*) and amino acid permease (*GmAAP6a*), which could induce amino acid transport from source to sink. Compared with CK, the inhibition of the above genes' expression by nitrogen deficiency (0N and LN) was more significant at 0N level than at LN level. Among them, under 0N treatment, *NR2, NiR, GS1*β, *GOGAT, GmAAP6a, PsaA, PsaF, PsbE*, and *PsbO* were downregulated by 5.75, 7.43, 4.67, 2.18, 5.67, 5.25, 4.68, 2.33, and 4.60 times, respectively, compared with the control. In contrast, when the seedlings under zero nitrogen stress were treated with MT, the expression of the above genes was only downregulated by 2.95, 6.20, 1.11, 0.79, 0.79, 3.36, 3.54, 1.83, and 3.67 times, respectively, in comparison with the CK.

### Effects of Exogenous Melatonin on Seed Yield and Seed Yield Components

**Figure 12:**
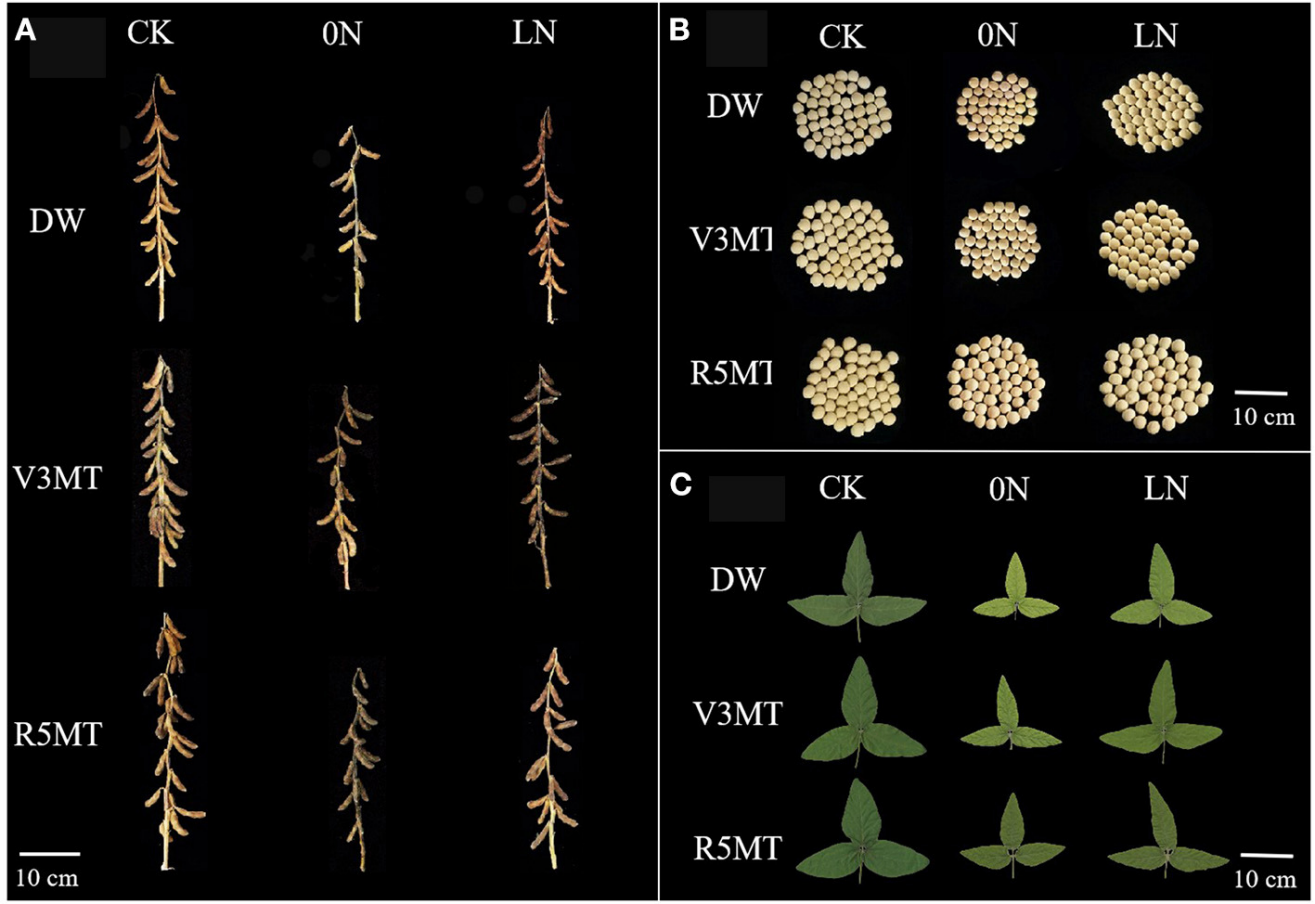
Effects of exogenous melatonin (V3 and R5) on the morphology of plant **(A)** and seed **(B)** at harvest, and the functional leaf morphology after 12 days of treatment **(C)**. CK, control nitrogen; 0N, zero nitrogen; LN, low nitrogen; DW, distilled water; V3MT, melatonin application at V3 stage; R5MT, melatonin application at R5 stage.

Compared with the CK, pod number, seed number, and seed weight individually increased by, respectively, 8.15, 9.75, and 8.74% in 2020 and by 10.86, 8.44, and 10.96% in 2021, when MT was applied alone in V3 stage (Figure 12 and Table 1). Insufficient nitrogen supply inhibited the yield of soybean, and the inhibitory effect of pod number, seeds number, grain weight, and 100-seed weight per plant of 0N were much more inhibited than LN when compared with CK. The negative effect of insufficient nitrogen supply on plant yield was alleviated with the use of MT in nitrogen-deficient plants. Among them, pod number, seed number, and grain weight of 0N+V3MT increased by 13.33, 16.55, and 16.69%, respectively, compared with 0N. Combined with the two-year production data to calculate the average efficiency, the above yield indices of LN+V3MT were 17.80, 15.21, and 12.41% higher than those of LN, respectively, and the number of pods and seeds returned to the level of CK treatment. The results showed that the application of MT at R5 stage increased the grain weight under 0N and LN levels by 3.07 and 3.22% (2-year average), respectively. Although the grain weight of LN+R5MT was 7.72% (2-year average) higher than that of LN, it was still lower than that of CK treatment.

**Table 1:** Effect of exogenous melatonin at V3 and R5 stages on soybean yield components under three nitrogen levels.

**Year**	**Treatment**	**Pod number per plant**	**Seed number per plant**	**Seed weight per plant (g)**	**100-seed weight (g)**
2020	CK	23.30, 1.06*b*	47.20, 1.47*b*	12.24, 0.28*b*	24.87, 0.26*a*
	V3MT	25.20, 1.03*a*	51.80, 1.75*a*	13.31, 0.36*a*	24.91, 0.12*a*
	R5MT	22.70, 1.46*b*	50.00, 2.37*ab*	12.25, 0.47*b*	24.97, 0.53*a*
	LN	16.60, 1.11*d*	40.10, 1.81*c*	9.75, 0.35*d*	23.57, 0.18*b*
	LN+V3MT	19.90, 0.78*c*	46.90, 2.20*b*	10.98, 0.26*c*	23.28, 0.32*b*
	LN+R5MT	17.60, 1.54*cd*	41.90, 2.86*c*	10.54, 0.26*c*	24.33, 0.24*a*
2021	CK	22.10, 2.98*bc*	47.40, 4.41*bc*	11.40, 0.79*b*	23.91, 0.26*abc*
	V3MT	24.50, 2.66*a*	51.40, 2.97*a*	12.65, 1.13*a*	23.53, 0.31*bc*
	R5MT	23.90, 1.81*ab*	49.50, 2.66*ab*	11.87, 1.05*ab*	24.39, 0.40*a*
	0N	15.00, 1.90*f*	29.00, 4.90*g*	7.37, 0.76*f*	22.77, 0.16*d*
	0N+V3MT	17.00, 2.77*e*	33.80, 3.82*f*	8.60, 0.87*e*	22.81, 0.31*d*
	0N+R5MT	14.60, 1.62*f*	31.70, 4.12*fg*	7.56, 0.53*f*	23.47, 0.17*c*
	LN	17.90, 1.64*e*	38.60, 3.69*e*	9.26, 0.79*de*	23.34, 0.18*cd*
	LN+V3MT	20.40, 1.80*cd*	43.80, 3.79*cd*	10.39, 0.85*c*	23.57, 0.43*bc*
	LN+R5MT	19.50, 1.91*de*	40.30, 4.03*de*	9.94, 0.71*cd*	24.10, 0.24*ab*

*The values are presented as mean ± SD (n=10). Different letters in one measuring group indicate statistically significant differences (P ≤ 0.05)*.

### Effects of Exogenous Melatonin on all the Parameters Studied Under Each Nitrogen Level

**Figure 13:**
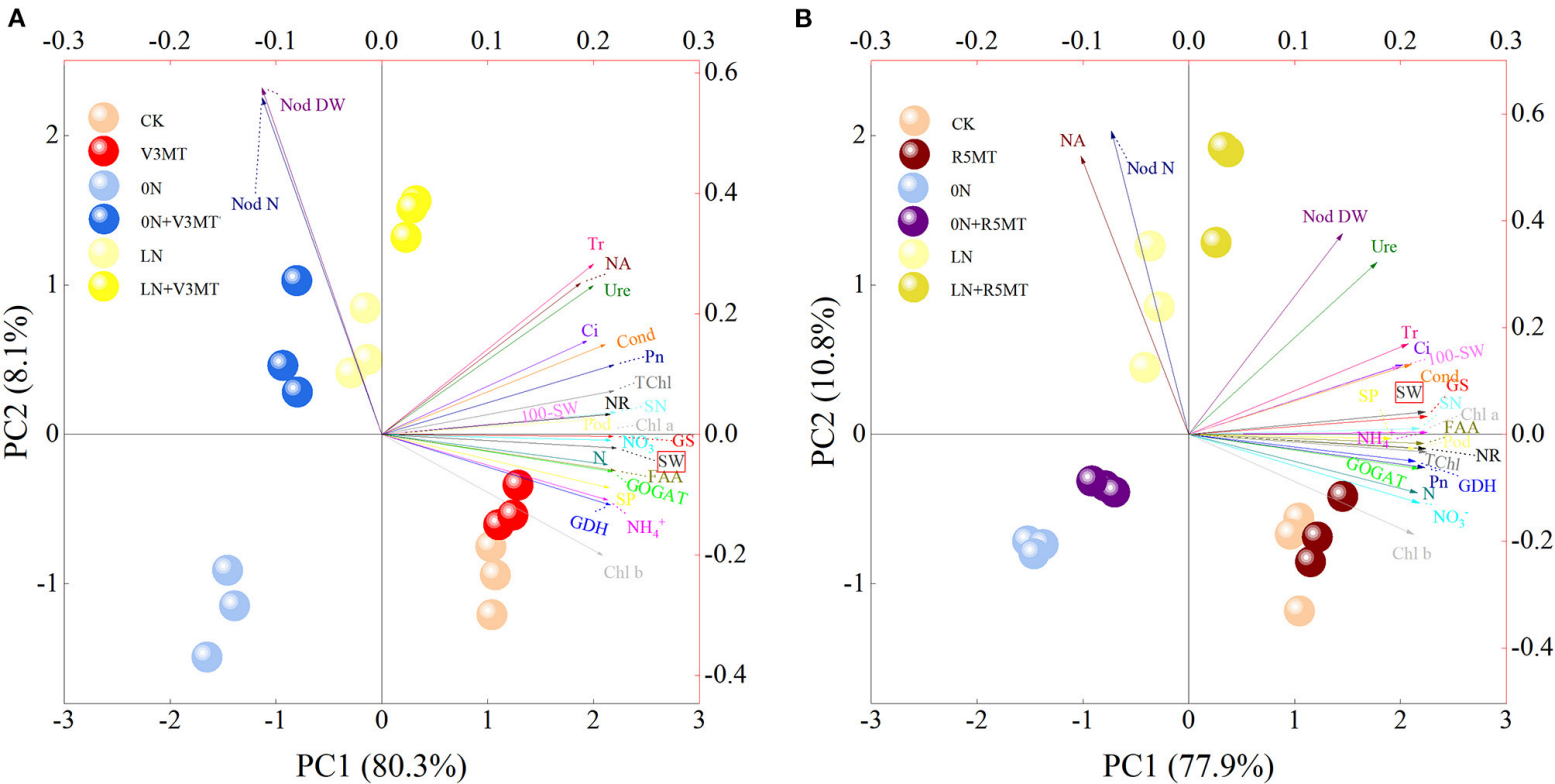
Principal components analysis (PCA) biplot showing the differences among treatments after melatonin spraying at V3 **(A)** or R5 **(B)** stage and the relationship among the related indicators that describe them. The relevant indicators are shown by arrows, while the different treatments are indicated as points. NR, Nitrate reductase; GS, glutamine synthetase; GOGAT, glutamate synthase; GDH, glutamate dehydrogenase; ${\text{NO}}_{3}^{-}$, nitrate; ${\text{NH}}_{4}^{+}$, ammonium; SP, soluble protein; FAA, free amino acid; Nod N, nodules number; Nod DW, nodules dry weight; NA, nitrogenase activity; Ure, ureide; N, nitrogen; Pn, net photosynthetic rate; Cond, stomatal conductance; Ci, intercellular carbon dioxide concentration; Tr, transpiration rate; Chl a, chlorophyll a; Chl b, chlorophyll b; TChl, total chlorophyll; Pod, pod number; GN, grains number; 100-SW, 100-seed weight; SW, seed weight. The abbreviated meaning of each treatment is the same as in Figure 3.

The PCA biplot of exogenous MT 12 days after V3 and R5 treatments explained the total variability by 88.4 and 88.7%, respectively (Figure 13). The effect of MT was analyzed based on the principal component 1 (PC1) that accounted for a larger proportion in the cause of differences among treatments. The score plot reveals that the distance between the points representing 0N+V3/R5MT and CK projected to the PC1 axis was less than that between 0N and CK. Similarly, the distance between the points representing LN+V3/R5MT and CK projected to the PC1 axis was smaller than that of LN and CK. MT treatment effectively reduced most of the differences in 0N or LN with CK due to nitrogen deficiency. In addition, the correlation between physiological characters and yield was analyzed by loading plot. All indices had a positive correlation with yield after MT treatment at V3 stage, except for the dry weight and the nodule number. Similarly, after MT treatment at R5 stage, while the correlation between yield and dry weight of nodule or nitrogenase activity decreased to almost negligible, the other indices were all significantly positively correlated with yield parameters.

## Discussion

Nitrogen metabolism is inhibited when plants first receive nitrogen-deficiency signals (Kubar et al., 2021). Qiao et al. (2019) confirmed that exogenous MT can effectively alleviate the stress of low nitrogen on wheat by enhancing the activities of NR and GS in leaves and maintaining high inorganic content as nitrogen metabolic substrate. In this study, we further explored the regulatory effect of MT on nitrogen metabolism in soybean under different nitrogen-deficiency levels. When compared with zero nitrogen treatment, the activity of NR was more significantly affected by exogenous MT at LN level. Combined with the change in intracellular nitrate content, the insufficient supply of nitrate reductase substrate under zero nitrogen conditions limited the ability of MT to promote nitrate absorption. However, under the condition of nitrogen deficiency, the activity of GS and its downstream ammonium assimilation enzymes were noticeably enhanced after MT treatment.

Glutamine synthetase plays a central role in the nitrogen metabolism of complex substrates as the major assimilatory enzyme for ammonia was produced from N fixation and nitrate or ammonia nutrition (Yin et al., 2022). It also re-assimilates ammonia released by photorespiration and the breakdown of proteins and nitrogen transport compounds (Miflin and Habash, 2002). This suggests that MT could improve the nitrogen use efficiency of nitrogen-deficient soybean by promoting the glutamate cycle. The substrate of GS is mainly derived from the metabolic process of nodule nitrogen fixation under zero nitrogen conditions (Carter and Tegeder, 2016). Therefore, the enhancement of ammonium absorption and metabolism induced by MT at nitrogen deficiency levels may also be attributed to its beneficial effect on root nodule development.

Root nodule nitrogen fixation is a unique way of acquiring nitrogen in legume crops. Soybean supplies dicarboxylic acids as a carbon source for rhizobia (Prell and Poole, 2006). In addition, the supply of branched-chain amino acids to bacteroids by plants is essential for development (Prell et al., 2009). In this study, we found that MT spray promoted the development of soybean nodules and determined that it was related to the production of more energy in plant carbon metabolism (Wei et al., 2015). This was attributed to the enhancement of nitrogen metabolism in the plant, which in turn promoted the synthesis of amino acids (Zou et al., 2021). Therefore, the strengthened nodule development may be due to MT causing plants to provide more abundant carbon sources and essential amino acids for root nodules. Ren et al. (2019a,b) soaked soybean seeds with MT and reported a similar conclusion that MT promotes root nodule development. The application of MT at V3 stage was more conducive to the number of nodules and the accumulation of dry matter, compared with the R5 stage. Since root nodule nitrogen fixation begins at the early vegetative stage, nodules fix nitrogen for 6–7 weeks until the plants reach the grain filling stage (R5), whereupon they begin to undergo senescence (Sanders, 2017). Undoubtedly, the effect of MT on the activity of N_2_ fixing bacteria and the specific regulatory mechanism need to be further determined by molecular biological methods.

Chlorophyll content is related to plant N nutrition, and it can be used as an indicator to detect the crop N nutrient conditions precisely and in a timely manner (Cendrero-Mateo et al., 2015). Notably, GS converts alpha-ketoglutarate and ammonia to glutamate that may serve as a nitrogen donor for the biosynthesis of chlorophylls (Miflin and Habash, 2002; Ferreira et al., 2019). Since soybean is a C3 plant, the beneficial effect of MT on chlorophyll synthesis may be related to the enhancement of NH_3_ re-assimilation produced by photorespiration under insufficient nitrogen conditions. However, the pathway needs to be further verified by isolating GS subtypes in future studies. Interestingly, MT promoted the synthesis of Chl a under low nitrogen condition, thus increasing the Chl a/Chl b value. In plants, the reaction centers of photosystems I and II are enclosed within core complexes that contain a precisely defined set of proteins that are encoded in the chloroplast genome. Compared with Chl b, the primary cofactor for the photochemical reactions in these complexes, Chl a is required for the assembly of these complexes (Eggink et al., 2001). The results indicate that MT can increase light energy absorption and promote photosynthesis and carbon assimilation by alleviating decreased Chl a level during nitrogen deficiency in soybean.

Under nitrogen deficiency, decreased photosynthetic pigment concentration in leaves was accompanied by altered Pn. In this study, the Cond, Ci, and Tr were clearly inhibited in soybean leaves without exogenous nitrogen supply. The factors for decreased photosynthetic capacity under 0N level also included stomatal limiting. MT had also been proved to be effective in alleviating photosynthetic damage caused by stomatal factors (Zou et al., 2021). This may explain the MT-enhanced photosynthesis of plants under zero nitrogen level. Furthermore, MT can improve light energy utilization efficiency and electron transfer ability through the expression of related genes in the upregulation of PSI and PSII and directly promote plant photosynthesis (Wei et al., 2015; Liang et al., 2019). In addition, MT treatment induces the transcription of genes related to PSI and PSII (*PsaA, PsaF, PsbE*, and *PsbO*). The high expression levels of these genes are conducive to the growth of the soybean plant (Wei et al., 2015). Moreover, enhanced photorespiration induces more NH_3_ re-assimilation to alleviate the damage caused by nitrogen deficiency (Ferreira et al., 2019). MT may also activate the organic acid pathway to produce more carbon skeleton needed for nitrogen assimilation, thereby enhancing nitrogen metabolism and alleviating the inhibitory effects of nitrogen deficiency on plant growth and development (Yanagisawa et al., 2004). However, it is necessary to further study the identification of elements related to this pathway.

Melatonin treatment is beneficial to maintain the integration and coordination of carbon and nitrogen metabolism to promote plant growth and restore yield (Erdal, 2019). Our previous studies have confirmed that MT application at the seedling stage is favorable to plant morphogenesis of soybean (Wang et al., 2021b). In this study, the grain weight per plant was observed to be higher with the application of MT at the critical stage of morphogenesis (V3) than that at grain filling (R5) under different nitrogen conditions. Wei et al. (2015) also demonstrated that MT-coated soybeans have higher yield potential. We observed that the application of MT at V3 stage promoted the yield mainly by increasing the number of pods and grains per plant, while the application at R5 stage mainly enhanced the grain weight. This phenomenon may be due to the spraying of MT during the critical period of morphogenesis to optimize vegetative growth, providing a better material basis for flowering and seed formation, thus increasing the yield.

**Figure 14:**
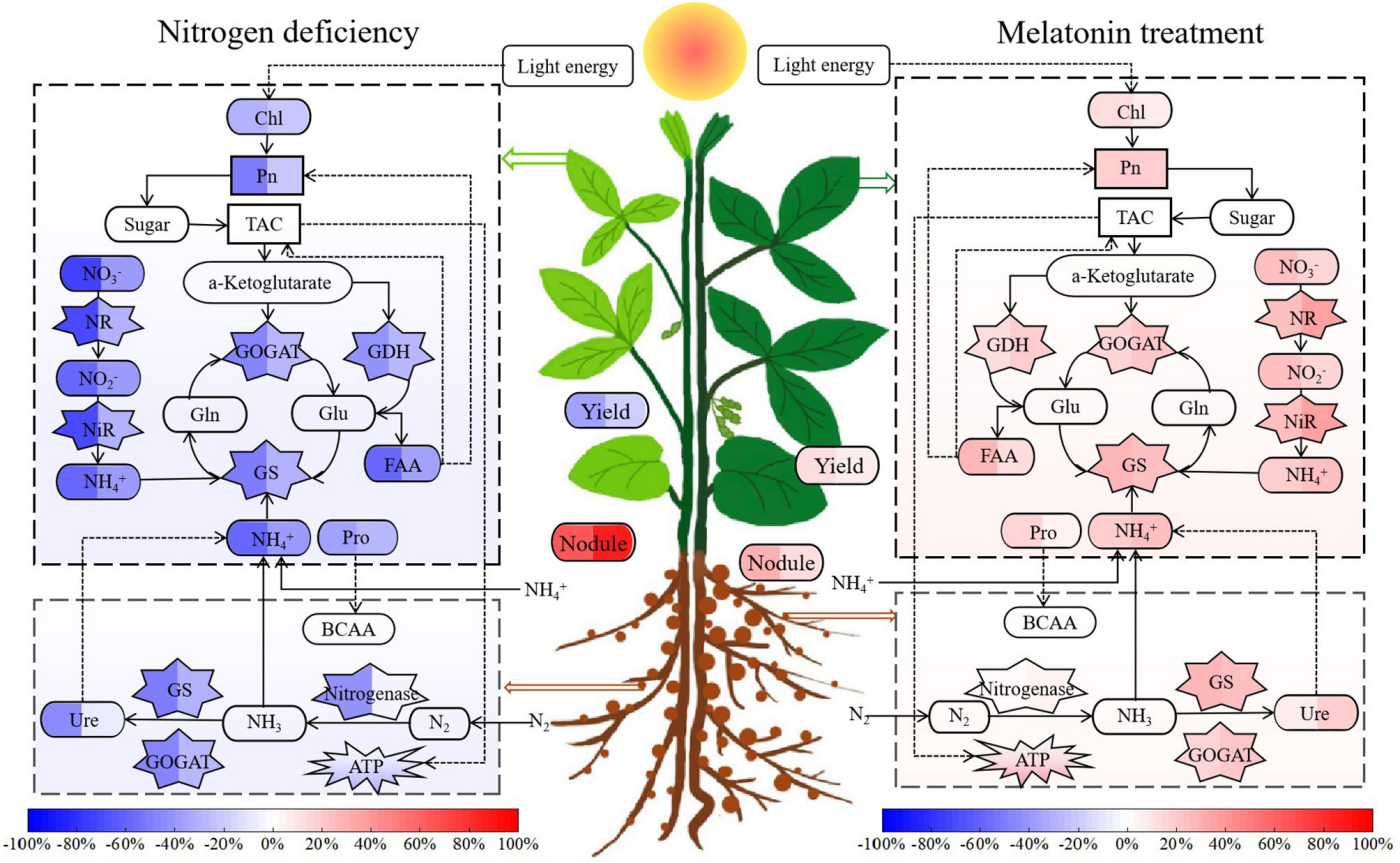
The left of the schematic model displays the changes in physiological metabolism and yield of soybean under 0N and LN treatments compared with the control. The left data in the heat map were taken from 0N and the right data were taken from LN, while the right of the schematic model shows the regulatory effect of MT application at V3 stage on carbon and nitrogen metabolism balance and yield compared with no MT treatment under 0N and LN supply levels. The left data in the heat map were taken from 0N+V3MT and the right data were taken from LN+V3MT. The blue depth indicates the degree of decrease, and the red depth indicates the degree of increase. TAC, tricarboxylic acid cycle; BCAA, branched-chain amino acid. The abbreviated meaning of other indices is the same as in Figure 13.

Overall, as shown in Figure 14, both 0N and LN treatments inhibited physiological metabolism and reduced biological yield in soybean, and the stress degree of nitrogen deficiency was positively correlated with the decrease in nitrogen supply. Under 0N level, the application of MT at V3 stage enhanced the nitrogen fixation ability of soybean mainly by strengthening nodule development, whereas at LN level, MT also promoted the activity of NR and accelerated the absorption of exogenous nitrate. Both provided more substrates for GS nitrogen assimilation, thus facilitating the synthesis and transport of amino acids. As another result of this, the increase in chlorophyll synthesis would induce enhancement of photosynthetic capacity by MT. The balance between carbon and nitrogen metabolism is the basis for plant development, and photosynthetic carbon metabolism (specifically the tricarboxylic acid cycle) can continuously provide a carbon skeleton for biological processes, including enzyme synthesis, activity regulation, and amino acid transport (Lawlor, 2002; Duan et al., 2018). Therefore, the direct stimulating effect of MT on the photosystem may alleviate the stress of altered carbon-nitrogen balance. The enhanced level of photosynthesis would eventually increase the accumulation of dry matter, which may provide a richer energy basis for grain filling at the yield formation stage, thus alleviating the yield limitation caused by nitrogen deficiency.

## Conclusion

The 0N or LN treatment significantly reduced the physiological and metabolic capacity and limited the yield of soybean. However, the dry matter accumulation of root nodules increased at three nitrogen levels after applying MT at two important growth stages. Particularly at V3 stage, the nodule number increased significantly, and the total nitrogen fixation capacity was also better with the MT application. MT enhanced the tolerance to nitrogen deficiency also by upregulating the expression of genes related to nitrogen metabolism (*NR2, NiR, GS1*β, *GOGAT*, and *GmAAP6a*) and promoting the activity of key enzymes (NR, GS, GOGAT, and GDH) in functional leaves, thus increasing the contents of amino acids, protein, and total nitrogen in plants. Exogenous MT was beneficial to the expression of photosynthesis-related genes, and it increased chlorophyll content to improve photosynthetic capacity. We further confirmed that the application of MT at V3 stage exhibited a better promoting effect on yield than at R5 stage, and the average grain weight per plant increased by 16.69, 12.20, and 10.96% under three nitrogen levels. Nevertheless, the MT-mediated resilience under nitrogen deficiency in soybean and its mechanism should be investigated further in depth, using molecular approaches.

## Data Availability Statement

The original contributions presented in the study are included in the article/Supplementary Material, further inquiries can be directed to the corresponding author/s.

## Author Contributions

YZ, GY, XJ, MW, and MZ contributed to the conception and design of the study. CR organized the database. LC and QZ performed the statistical analysis. HW wrote the first draft of the manuscript. All authors contributed to manuscript revision, read, and approved the submitted version.

## Funding

This study was financially supported by the China Agriculture Research System of MOF and MARA (No. CARS-04-PS18), the Heilongjiang Province Revealing the List and Commanding the Leader Science and Technology Project (No. 2021ZXJ05B02), the Heilongjiang Postdoctoral Scientific Research Startup Program (Fund Program for Overseas Returnees, No. LBH-Q20052), the Initiation Foundation for Heilongjiang Bayi Agricultural University Support Program for San Heng San Zong (No. ZRCQC202101), the Heilongjiang Bayi Agricultural University Support Program for San Heng San Zong (No. TDJH202001), the Research and Demonstration Project of Key Technology of Adzuki Bean Mechanization and High Yield in Daqing (No. zd-2021-81), and the Research Initiation Plan for Talent Introduction (No. XYB202011).

## Conflict of Interest

The authors declare that the research was conducted in the absence of any commercial or financial relationships that could be construed as a potential conflict of interest.

## Publisher's Note

All claims expressed in this article are solely those of the authors and do not necessarily represent those of their affiliated organizations, or those of the publisher, the editors and the reviewers. Any product that may be evaluated in this article, or claim that may be made by its manufacturer, is not guaranteed or endorsed by the publisher.
