# Supplementary material for: Exogenous Melatonin Modulates Physiological Response to Nitrogen and Improves Yield in Nitrogen-Deficient Soybean (Glycine max L. Merr.)
Source: Front Plant Sci. 2022 May 16;13:865758. doi: 10.3389/fpls.2022.865758 (PMC9149585; doi:10.3389/fpls.2022.865758)
Supplement: Supplementary file 2 [file Image_2.pdf]

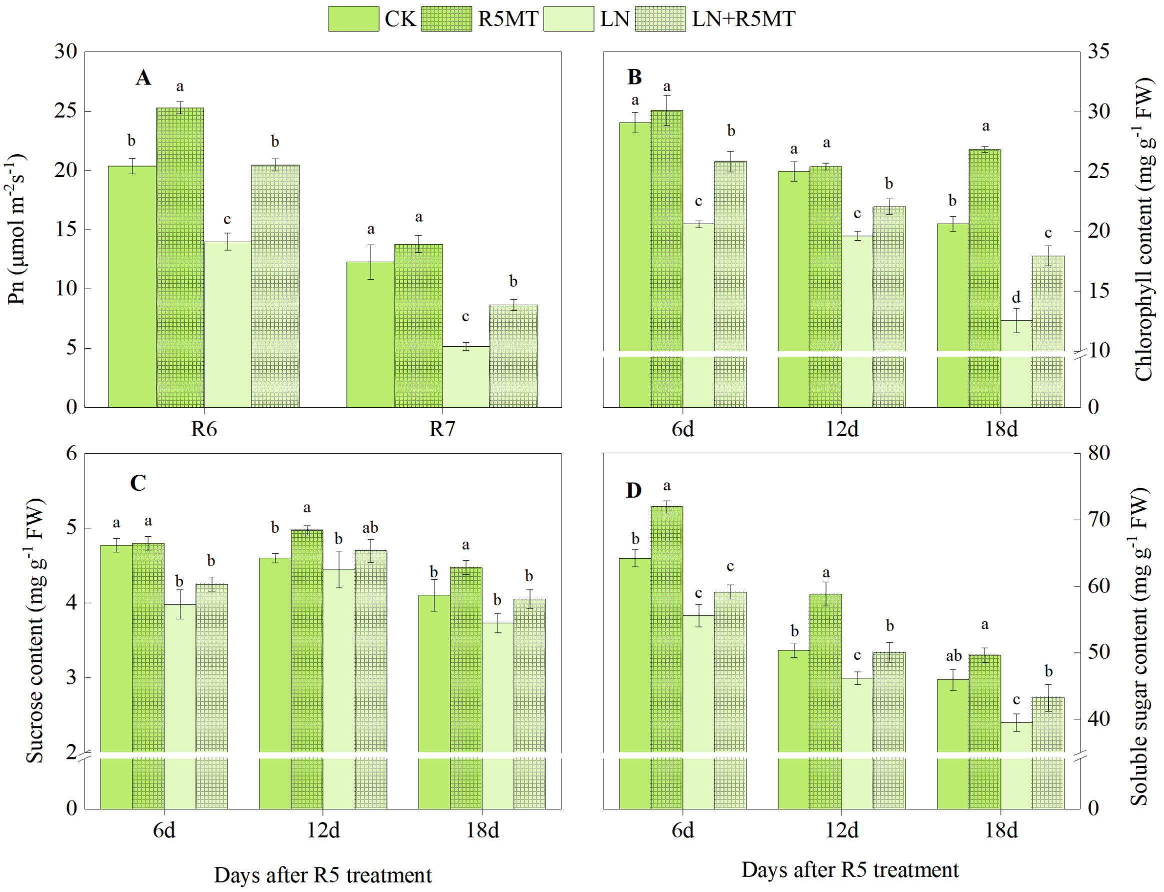

Supplementary Figure 2. Effects of spraying melatonin at R5 stage on net photosynthetic rate (Pn, **A**), chlorophyll content (**B**), sucrose content (**C**) and soluble sugar content (**D**). The abbreviated meaning of each treatment is the same as in Fig 2. The values were presented as mean ± SE (n=3). Different letters in one measuring group indicate statistically significant differences when $P \leq 0.05$.
