# Supplementary material for: Exogenous Melatonin Modulates Physiological Response to Nitrogen and Improves Yield in Nitrogen-Deficient Soybean (Glycine max L. Merr.)
Source: Front Plant Sci. 2022 May 16;13:865758. doi: 10.3389/fpls.2022.865758 (PMC9149585; doi:10.3389/fpls.2022.865758)
Supplement: Supplementary file 4 [file Table_2.doc]

| **Supplementary Table 2.** Effect of spraying melatonin at V3 and R5 stages on soybean dry matter under different nitrogen levels | | | | | |
| --- | --- | --- | --- | --- | --- |
| Sampling stage | Treatment | Dry weight of  above ground/ g | Dry weight of nodules/ g | Dry weight of root / g | Root- top ratio / % |
| V5 | CK | 1.93±0.15 a | 0.037±0.005 d | 0.89±0.06 a | 46.22±3.74 b |
|  | CN+V3MT | 1.79±0.02 a | 0.057±0.005 c | 0.99±0.09 a | 55.42±4.99 ab |
|  | LN | 1.76±0.18 a | 0.107±0.009 b | 0.87±0.06 a | 49.88±1.63 ab |
|  | LN+V3MT | 1.65±0.07 a | 0.197±0.009 a | 0.97±0.03 a | 58.67±2.78 a |
| R1 | CK | 2.52±0.06 b | 0.070±0.008 c | 1.11±0.03 b | 44.20±1.45 c |
|  | CN+V3MT | 3.12±0.24 a | 0.083±0.012 c | 1.46±0.06 a | 46.93±4.22 c |
|  | LN | 2.01±0.18 c | 0.107±0.005 b | 1.08±0.12 b | 53.88±1.19 b |
|  | LN+V3MT | 2.40±0.11 b | 0.220±0.008 a | 1.46±0.07 a | 60.96±3.80 a |
| R3 | CK | 15.67±0.14 bc | 0.257±0.012 d | 5.30±0.29 b | 33.85±1.99 c |
|  | CK+V3MT | 17.99±0.43 a | 0.303±0.012 c | 5.99±0.22 a | 33.28±0.44 c |
|  | LN | 13.05±0.69 d | 0.487±0.005 b | 5.42±0.48 ab | 41.49±1.57 a |
|  | LN+V3MT | 14.97±0.33 c | 0.553±0.025 a | 5.66±0.17 ab | 37.79±0.36 b |
| R5 | CK | 19.12±0.09 b | 0.917±0.049 d | 6.10±0.53 b | 31.88±2.64 b |
|  | CK+V3MT | 21.60±0.29 a | 1.077±0.021 c | 6.71±0.33 b | 31.06±1.26 b |
|  | LN | 16.89±0.68 c | 1.490±0.051 b | 6.89±0.17 ab | 40.83±0.97 a |
|  | LN+V3MT | 18.35±0.61 b | 1.740±0.054 a | 7.69±0.35 a | 41.92±1.23 a |
| R6 | CK | 20.91±0.46 b | 0.930±0.029 d | 6.67±0.38 b | 32.31±1.01 b |
|  | CK+V3MT | 24.60±1.63 a | 1.033±0.041 c | 7.13±0.23 b | 29.05±1.27 b |
|  | LN | 18.34±0.57 c | 1.657±0.039 b | 7.09±0.29 b | 38.70±1.97 a |
|  | LN+V3MT | 19.55±0.69 bc | 1.973±0.074 a | 8.11±0.18 a | 41.58±2.11 a |
| R7 | CK | 23.11±0.86 b | 0.903±0.031 e | 7.94±0.06 c | 34.41±1.36 b |
|  | CK+V3MT | 24.81±0.33 a | 1.147±0.094 d | 8.20±0.05 bc | 33.06±0.24 b |
|  | CK+R5MT | 22.62±0.84 bc | 1.127±0.062 d | 7.92±0.26 c | 35.01±0.19 b |
|  | LN | 17.93±0.37 d | 1.627±0.026 c | 8.06±0.20 c | 44.94±1.42 a |
|  | LN+V3MT | 21.28±0.82 c | 2.037±0.071 a | 9.03±0.09 a | 42.47±1.37 a |
|  | LN+R5MT | 19.32±0.94 d | 1.790±0.041 b | 8.63±0.38 ab | 44.75±2.48 a |
| R8 | CK | 23.76±0.84 b | 0.900±0.054 e | 8.07±0.25 c | 34.02±1.60 b |
|  | CK+V3MT | 26.09±0.41 a | 1.110±0.067 d | 9.12±0.36 ab | 34.98±1.90 b |
|  | CK+R5MT | 23.13±1.01 b | 1.040±0.079 d | 8.20±0.12 c | 35.54±1.75 b |
|  | LN | 19.87±0.91 c | 1.593±0.050 c | 8.06±0.20 c | 40.68±2.90 a |
|  | LN+V3MT | 22.50±0.94 b | 1.957±0.061 a | 9.41±0.51 a | 41.89±2.62 a |
|  | LN+R5MT | 20.43±0.54 c | 1.770±0.049 b | 8.60±0.38 bc | 42.17±2.77 a |
| The values are presented as mean ± SD (n=3). Different letters in one measuring group indicate statistically significant differences ( P ≤ 0.05). | | | | | |
